# Use of an Oral Device in the Stabilization of Facial Advancement after Early Removal of the Osteodistraction Device for Postoperative Sequelae

**DOI:** 10.3390/dj8010012

**Published:** 2020-01-19

**Authors:** Giulio Gasparini, Gianmarco Saponaro, Michela Perina, Roberto Boniello, Camillo Azzuni, Enrico Foresta, Giuseppe D’Amato, Mattia Todaro, Piero Doneddu, Luca Massimi, Gianpiero Tamburrini, Sandro Pelo, Umberto Garagiola, Alessandro Moro

**Affiliations:** 1 Maxillofacial Surgery Unit, Fondazione Policlinico Universitario A. Gemelli IRCSS, Catholic University Medical School, 00168 Rome, Italy; drgiuliogasparini@gmail.com (G.G.); gianmarco.saponaro@gmail.com (G.S.); perina.michela@gmail.com (M.P.); rboniello9@gmail.com (R.B.); camillo.azzuni@policlinicogemelli.it (C.A.); e.foresta@yahoo.it (E.F.); dottgdamato@gmail.com (G.D.); mattia.todaro@gmail.com (M.T.); sandro.pelo@unicatt.it (S.P.); mrolsn@libero.it (A.M.); 2 Paediatric Neurosurgery Unit, Fondazione Policlinico Universitario A. Gemelli IRCSS, Catholic University Medical School, 00168 Rome, Italy; luca.massimi@policlinicogemelli.it (L.M.); gianpiero.tamburrini@unicatt.it (G.T.); 3 Department of Biomedical, Surgical and Dental Sciences, School of Dentistry, University of Milan, 00168 Milan, Italy; umberto.garagiola@unimi.it

**Keywords:** craniofacial advancement, craniostenosis, oral device, relapse, osteodistraction

## Abstract

The aim of this study is to present an oral device that improves splanchnocranium stability after osteodistraction in children treated for correction of craniofacial malformations. When removal of the distraction device before the end of the treatment is necessary, the reposition of a new fixation system might not be possible. In these cases, regrown bone is immature, and relapse of malformation occurs frequently. We have been treating these cases by the application of an oral device named Maxillary Advancement Contention (MAC). MAC is used in every patient when any complication interrupts the protocol of osteodistraction before the end of the stabilization time. The device is placed immediately after the removal of the distraction device and left in place for at least three months. We used MAC in six children surgically treated for correction of craniosynostosis with facial or craniofacial advancement. To establish the relapse of malformation we analyzed relations Sella-Nasion-Orbitale (SNOr) and Sella-Nasion-A point (SNA) angles before application of the MAC and after one year. The analysis of stability was excellent in every patient. This device might help, with a minimally invasive procedure, to maintain the obtained advancement allowing stabilization of the regrown bone.

## 1. Introduction

The risk of relapse in children undergoing craniofacial advancement is very high. Due to the absence of rigid internal fixation systems this risk can increase significantly in those cases that need osteodistraction.

Our standard protocol for treating these cases provides a stabilization period of 120 days after the last activation of the osteodistraction device [1,2,3].

When removal of the distraction device before the end of the treatment is necessary, the reposition of a new fixation system might not be possible. In these cases newly formed bone is immature and relapse of malformation can frequently occur.

In this study we propose the use of an oral device that retains the splanchnocranium in a specific position in order to stabilize facial skeletal structures.

## 2. Materials and Methods

**Figure 1:**
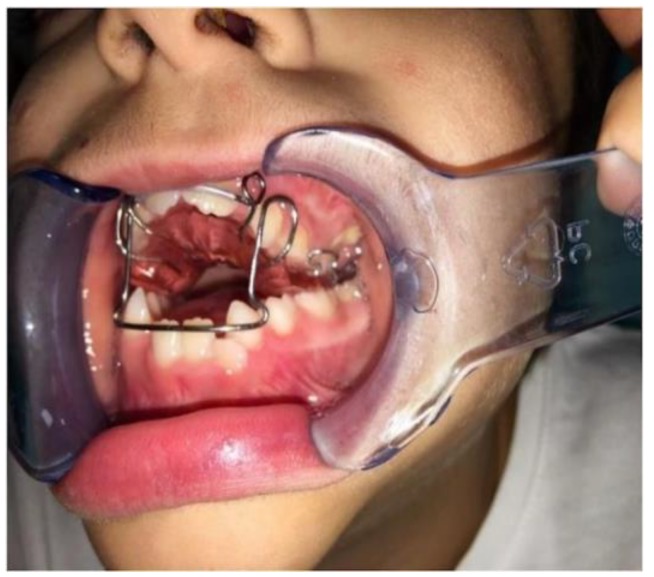
Maxillary Advancement Contention (MAC) Device: it is a functional activator with dental retention.

We called the oral device “Maxillary Advancement Contention (MAC)” [4]. (See Figure 1).

It consists of a functional activator with dental retention. It has an Adam’s hook on the last erupted dental elements on both sides and an occlusal plane in acrylic resin to prevent eruption and medialization of the lower primary teeth. A vestibular arch is located on its frontal side in order to stop the advancement of the lower incisors.

The MAC is placed immediately after the removal of the distraction device and left in place for at least 3 months.

In those cases where the distraction device has to be removed before the end of the stabilization, we suggest applying the device every day and removing it only for eating in the first three months. After this time, we recommend wearing it only at night for three more months.

Patients who completed osteodistraction treatment should wear the MAC only at night.

We used MAC in 6 patients, 4 females and 2 males, age ranging from 38–70 months, average 53 months. Two patients were affected by Apert’s Syndrome and 4 patients by Crouzon’s Syndrome. (See Table 1).

In order to evaluate the relapse of malformation we analyzed the splanchnocranium position on the cephalometric analysis measuring Sella-Nasion-Orbitale (S-N-Or) and Sella-Nasion-A point (S-N-A) angles with Dolphin v. 9.0 (Patterson Dental Supply, St Paul, MN, USA). We considered lateral cephalograms obtained after removal of device (T0) and after 1 year from the end of treatment with MAC (T1).

Patients are informed that in the event of complications, a device will be used to maintain the desired position of the splanchocranium and sign an informed consent before undergoing surgery. This device has the same purpose as the bone stabilizer.

## 3. Results

Patient 1 underwent Fronto-orbital advancement with Rigid Internal Fixation stabilization and Osteodistraction of splanchnocranium. In patient 2 and 6 we performed a Le Fort III osteotomy while other patients were treated with Monobloc advancement.

**Figure 2:**
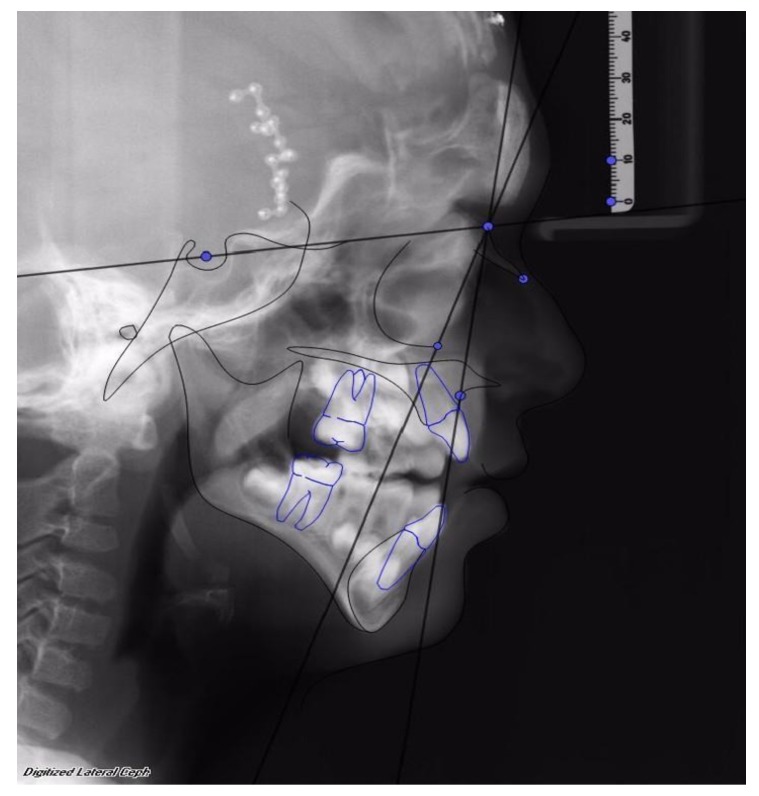
First patient, female, 6 years old, affected by Crouzon’s Syndrome. Osteodistraction (OD) was removed after 40 days before the established time of osteodistraction for fractures of bone support caused by accidental trauma. Osteodistraction was finished with an advancement of 23 mm. The advancement was interrupted before the normalization of the SNA angle to stop bone advancement. After removal of the OD device, the SNA was 75° and the SNOr was 63°.

**Figure 3:**
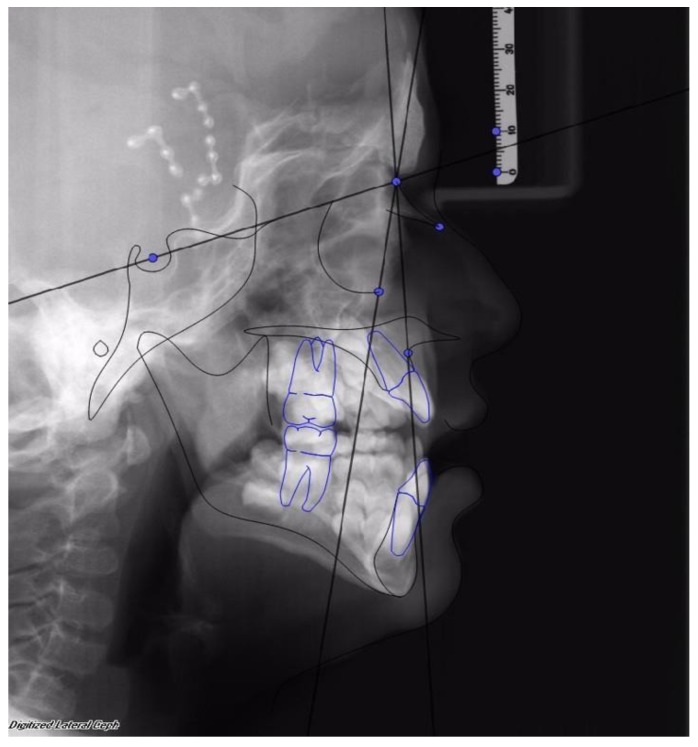
The same patient after 1 year from the end of treatment with MAC. SNA was 76° and SNOr was 63°.

Facial advancement was satisfying for every patient, only the advancement of patient 1 should have been greater in order to reach the appropriate projection. Clinical results were anyway satisfying, so no further surgical procedure was undertaken. (See Figure 2 and Figure 3).

In four patients the distractor was removed before the planned time due to complications arising during the bone stabilization phase. In these patients the distraction device was removed between the 32nd and the 64th day (average of 44–75 days). In two patients the Osteodistraction Device was removed for infectious complications and in another two for fractures of the underlying bone.

We used MAC in other two patients at the end of the osteodistraction phase to improve bone stability.

The follow up varied between 13 and 52 months, with an average of 32.2 months.

Bone stability was evaluated at the end of treatment and has been shown to be excellent for each patient. In every patient we observed an improvement of maxillary projection and in patients 2, 4, 5, and 6 we detected an advancement of the Orbitale (Or) position. (See Table 1).

## 4. Discussion

In children with facial-craniosynostosis functional complications might force anticipation of the time of craniofacial advancement [4,5,6,7,8].

In these cases, the early age at surgery increases the risk of anticipated removal of the osteodistraction device thus making the use of MAC appropriate.

We found several advantages in the use of MAC.

With a minimally invasive procedure the MAC system might help to maintain the achieved advancement allowing the stabilization of the newly formed bone.

The MAC keeps the splanchnocranium in the desired position. It can be removed if necessary. The management of the device is very easy and so is its removal and cleaning. These features, along with the reduced size of the device, increased patients’ compliance.

The main disadvantage of MAC is due to its manufacturing costs.

The effects of MAC on the temporo-mandibular joint (TMJ) have not been well investigated but due to its limited time of use, it is unlikely that it would cause any problem.

The MAC device can help to stabilize the position of the splanchnocranium but it is useless in maintaining the projection of the fronto-orbital region. In our study only one patient required stabilization of the frontal region with Rigid Internal Fixation, in the other cases we did not detect any relapse of the fronto-orbital bandeau but this can be due to the low number of patients. However, we suppose that the volume expansion of the brain could help to keep the achieved position of the upper third of the face.

The MAC does not provide a fixed retention but a dynamic retention, although it has not shown any negative effect on the formation and stabilization of the bone callus.

According to our clinical observations, the use of MAC must be extended up to six months. This time appears to be enough to allow the formation of a stable bone callus. Longer time of use could be useless and dangerous for the TMJ function and could lead to alteration of the normal development of the lower third of the face or dental eruption.

## 5. Conclusions

MAC is an effective aid to help bones to maintain the new position in cases of early removal of the stabilization system in patients treated with craniofacial osteodistraction. The device is easy to produce, not unduly expensive, compliance has been shown to be high, and in addition it gives us the required stability in order not to lose the achieved results.

To conclude our study demonstrates high efficiency for the use of MAC.

However, given the small size of our sample, more accurate studies will have to be carried out.

In particular the effects of the device on the TMJ should be investigated as well as the results on completion of the patient’s skeletal growth.

However, treated patients are being continuously followed up during their growth in order to evaluate the effects of the treatment in the long term.

## Figures and Tables

**Table 1:** Children treated with Osteodistraction protocol for therapy of craniofacial malformations. In patients 1, 2, 3, 4 the OD (OsteoDistraction) was removed before the end of bone stabilization because of severe complications. The OD was substituted with MAC. Patients 5 and 6 used MAC at the end of the stabilization time to improve the consolidation of callus bone. This table describes the splanchnocranium position before application of MAC and after 1 year.

PZ	Sex	Age in Months	Pathology	Surgery	Advancement in mm	Complication in OD	Stabilization Time in days	Cause of Removal Distraction Device	SNA T0	SNA T1	SNOr T0	SNOr T1	Follow Up (Months)
1 (SE)	F	70	Crouzon	Fronto-orbital advancement + LeFort III	23	Stop of advancement	40	fracture bone support	75	76	63	63	13
2 (LC)	F	60	Crouzon	Le Fort III	22	no	64	infection	80	81	68	69	22
3 (AA)	M	49	Crouzon	Monobloc	22	no	43	infection	78	79	70	70	45
4 (KS)	M	38	Apert	Monobloc	20	no	32	fracture bone support	80	81	72	73	34
5 (GC)	F	49	Crouzon	Monobloc	25	no	120	stabilization completed	80	81	68	70	52
6 (CF)	F	52	Apert	Le Fort III	21	no	120	stabilization completed	81	82	70	71	48
